# The diversity of the Chagas parasite, *Trypanosoma cruzi*, infecting the main Central American vector, *Triatoma dimidiata*, from Mexico to Colombia

**DOI:** 10.1371/journal.pntd.0005878

**Published:** 2017-09-28

**Authors:** Patricia L. Dorn, Annie G. McClure, Meghan D. Gallaspy, Etienne Waleckx, Adrienne S. Woods, Maria Carlota Monroy, Lori Stevens

**Affiliations:** 1 Loyola University New Orleans, New Orleans, Louisiana, United States of America; 2 Universidad Autónoma de Yucatán, Merida, Mexico; 3 University of San Carlos of Guatemala, Guatemala City, Guatemala; 4 University of Vermont, Burlington, Vermont, United States of America; Universidade Federal de Minas Gerais, BRAZIL

## Abstract

Little is known about the strains of *Trypanosoma cruzi* circulating in Central America and specifically in the most important vector in this region, *Triatoma dimidiata*. Approximately six million people are infected with *T*. *cruzi*, the causative agent of Chagas disease, which has the greatest negative economic impact and is responsible for ~12,000 deaths annually in Latin America. By international consensus, strains of *T*. *cruzi* are divided into six monophyletic clades called discrete typing units (DTUs TcI-VI) and a seventh DTU first identified in bats called TcBat. TcI shows the greatest geographic range and diversity. Identifying strains present and diversity within these strains is important as different strains and their genotypes may cause different pathologies and may circulate in different localities and transmission cycles, thus impacting control efforts, treatment and vaccine development. To determine parasite strains present in *T*. *dimidiata* across its geographic range from Mexico to Colombia, we isolated abdominal DNA from *T*. *dimidiata* and determined which specimens were infected with *T*. *cruzi* by PCR. Strains from infected insects were determined by comparing the sequence of the 18S rDNA and the spliced-leader intergenic region to typed strains in GenBank. Two DTUs were found: 94% of infected *T*. *dimidiata* contained TcI and 6% contained TcIV. TcI exhibited high genetic diversity. Geographic structure of TcI haplotypes was evident by Principal Component and Median-Joining Network analyses as well as a significant result in the Mantel test, indicating isolation by distance. There was little evidence of association with TcI haplotypes and host/vector or ecotope. This study provides new information about the strains circulating in the most important Chagas vector in Central America and reveals considerable variability within TcI as well as geographic structuring at this large geographic scale. The lack of association with particular vectors/hosts or ecotopes suggests the parasites are moving among vectors/hosts and ecotopes therefore a comprehensive approach, such as the Ecohealth approach that makes houses refractory to the vectors will be needed to successfully halt transmission of Chagas disease.

## Author summary

Little is known about the strains of the Chagas parasite circulating in Central America. This parasite is responsible for the most serious parasitic disease in Latin America and is presently divided into seven different strains. In Central America, the Chagas parasite is spread mainly by one species of kissing bug but what strains are present in this species was largely unknown. We investigated which strains of the parasite are present in this species of kissing bug by examining the DNA extracted from the abdomens of kissing bugs collected across its geographic range. We matched the DNA sequence we obtained with what is available in databases to determine the strain. We found mostly strain TcI (94%) and less of strain TcIV (6%). As particular strains are associated with particular habitats, hosts, and disease symptoms, this work will help us understand why particular symptoms occur in particular areas and help us to target control efforts more efficiently.

## Introduction

*Trypanosoma cruzi* is the causative agent of Chagas disease and infects approximately six million people in Latin America [1] as well as many other mammals. Although initially considered largely clonal with only rare genetic mixing during its evolution [2], studies of the population structure and increasingly detailed studies of the nuclear and mitochondrial DNA of *T*. *cruzi* clones have suggested that this clonal propagation is overlaid with more frequent and recent hybridization and genetic exchange events than was previously appreciated [3]. Six discrete-typing units (DTU TcI-VI) are currently recognized, adopted by international consensus [4], including at least two hybrid lineages (TcV and TcVI), and one additional lineage found mostly in bats (TcBat) [5, 6], which is closely related to TcI. Among these DTUs, TcI is the most widespread and diverse lineage, with the smallest genome and the least amount of aneuploidy so is likely a parent of some hybrid lineages [4].

Knowledge of the *T*. *cruzi* strains present will be useful for understanding the epidemiology, and for treatment and control as different strains are roughly associated with different geographic locations and ecotopes, and hosts and vectors (reviewed in [7]). Association of particular strains with the diverse disease spectrum observed is an area of intense investigation [7, 8]. For example, the megasyndrome form of Chagas disease is found mostly in Southern Cone countries where TcV and TcVI are found in humans, whereas cardiomyopathy, rather than megasyndrome, is common in Central and North America where TcI is associated strongly with human infection [7]. Although direct association of particular disease spectrum and specific *T*. *cruzi* DTU remains elusive [7], some recent studies are beginning to dissect the mechanisms and show that at least some of different disease spectra are likely due to different *T*. *cruzi* strains [8]. In addition, the particular strains involved in human infections should also be a consideration for treatment efficacy studies and drug design [9].

TcI is found from the southern U.S. to southern South America and was first associated with sylvan cycles (marsupials and rodents) in South America (reviewed in [4]). Later studies showed that TcI is the most common strain identified in northern South America [10, 11], as well as Central and North America and is frequently associated with human disease from the Brazilian Amazon basin northwards [11–18]. TcI is considered more diverse (and therefore originating) in South America compared to North and Central America [19]. However, the higher diversity in the south as compared to the north could reflect the relative geographic range surveyed and/or the sampling effort as little *T*. *cruzi* strain typing has been reported other than South America [20]. In addition, although most strains found in humans are reflective of the strains found in nearby hosts and vectors [7], there is an intriguing divergent, and fairly homogenous TcI subgroup associated with human infections (now called TcI_DOM_) [19, 21, 22]. Identified to date largely in South America and surprisingly distinct from strains found in nearby hosts or vectors; TcI_DOM_ clusters with North and Central America strains by phylogenetic inference [19].

A recent inventory notes that of the DTUs published for *T*. *cruzi* isolates, 90.7% are from South America; little is known about strains present in Central and North America [20]. Even less is known about *T*. *cruzi* DTUs present in *Triatoma dimidiata*, the principal Chagas vector in Central America and a secondary vector in Mexico and northern South America. Of limited studies from this geographic region, most report the predominance of TcI and less frequent presence of TcIV [12, 14, 15, 17, 18, 23–25]. A broader range of DTUs has also been reported in Mexico [26, 27].

The purpose of this study was to determine the strains of *T*. *cruzi* present in *T*. *dimidiata*, across its broad geographic range from southern Mexico to northern South America [28], by comparing the sequence of two nuclear markers: 18S rDNA and the spliced leader, also known as mini-exon, intergenic region (SL-IR) to that of strains of known DTU. In addition, we explore how adding the largest sample of Central American *T*. *cruzi* strains to date informs the diversity present within TcI. We investigate clustering of TcI haplotypes by geography, host and ecotope using Principle Component and Median-Joining Network analyses. This study provides new information describing the diversity of *T*. *cruzi* circulating in *T*. *dimidiata* from Mexico to Colombia, and relates this TcI diversity to that found elsewhere in the Americas.

## Materials & methods

### Specimen collection

A total of 334 adult *T*. *dimidiata* were collected from 19 sites in eight countries across the geographic range of the species, from Mexico to Ecuador, by professionals trained in safe handling of biohazardous materials (S1 Table). Specimens were collected by the person/hr method inside homes (domestic), in areas surrounding homes (peridomestic) or in sylvan areas. Specimens were stored at -20°C in a 95% ethanol / 5% glycerol until DNA was isolated. DNA from reference strains was kindly provided by Drs. Christian Barnabé and Frédérique Brenière (IRD, France). A TcI reference strain, Silvio X10 was purchased from ATCC (Manasses, VA).

### *T*. *cruzi* detection and strain determination

DNA was isolated from the distal two abdominal segments of *T*. *dimidiata* specimens exactly as specified in the DNeasy Blood and Tissue kit (Qiagen, Inc., Valencia, CA). Infection of the *T*. *dimidiata* specimens with *T*. *cruzi* was determined by PCR (AmpliTaq DNA polymerase, Life Technologies, Grand Island, NY) using the TCZ1 and TCZ2 primers [29] and these cycling conditions: an initial denaturation at 94°C for 10 min; 30 cycles of 94°C for 20 sec, cooling to 57°C for 10 sec, and heating to 72°C for 30 sec, and final extension for 7 min at 72°C. Amplified products were electrophoresed alongside a positive control of amplified *T*. *cruzi* DNA and a negative control of water on a 1.8% agarose gel containing DNA SafeStain (Lambda Biotech, Inc., St. Louis, MO) and visualized on a UV transilluminator (Bio Rad, Hercules, CA). The PCR was repeated if the controls did not give the expected results.

*T*. *cruzi* strains present in *T*. *cruzi-*positive *T*. *dimidiata* specimens were determined by amplification and sequencing of 18S rDNA and the SL-IR; these two nuclear genomic regions can distinguish all six *T*. *cruzi* DTUs [4]. 18S rDNA was amplified using V1 and V2 primers [30] and these cycling conditions: initial denaturation at 94°C 2 min, followed by 30 cycles of: 94°C 1 min, 54°C 1 min, 72°C 1 min, and a final step of 72°C for 5 min. SL-IR was amplified using Tc, Tc1, and Tc2 primers together [30] and these cycling conditions: initial denaturation 94°C 2 min, followed by 27 cycles of 94°C 30 sec, 55°C 30 sec, 72°C 30 sec, and a final step at 72°C 5 min. PCR products were electrophoresed on a 2% MetaPhor Agarose (Cambrex Bio Science Rockland, Inc., Rockland, ME, USA) and visualized by transillumination.

Eighty-two *T*. *dimidiata* abdominal DNA samples amplified at the expected band size for their respective marker [4] and were sequenced (Beckman Coulter Genomics, Danvers, MA, USA). 18S sequence was determined for 44 specimens by direct sequencing or sequencing following cloning if overlapping peaks were observed in the chromatogram (12 specimens, p-GEM-T easy vector system, Promega, Madison, WI, USA, Table 1). SL-IR sequence was determined for 22 specimens by direct sequencing. Sequence for both markers was obtained for 15 specimens, which allowed us to determine the *T*. *cruzi* strain in 51 individual specimens. *T*. *cruzi* strains were unambiguously determined for each sequence based on ≥97% query coverage and ≥98% identity to a published strain (DTU) in a Blast query (http://blast.ncbi.nlm.nih.gov/Blast.cgi)).

**Table 1:** Strains (DTUs) of *Trypanosoma cruzi* in *Triatoma dimidiata* across its geographic range.

No.	Collection Site	Specimen ID	18S rDNA	SL-IR
1	**Belize**	Bz01	TcIV*	ND
2	Calla Creek, Cayo District, **Belize**	BzCaCC05	TcI*	ND
3	Calla Creek, Cayo District, **Belize**	BzCaCC07	TcI*	ND
4	Calla Creek, Cayo District, **Belize**	BzCaCC09	TcI	ND
5	Calla Creek, Cayo District, **Belize**	BzCaCC10	TcI*	TcI*
6	Calla Creek, Cayo District, **Belize**	BzCaCC13	TcI	ND
7	Calla Creek, Cayo District, **Belize**	BzCaCC14	TcI	TcI
8	San Jose, Toledo District, **Belize**	BzTDSJ01Cl1	TcI	ND
9	San Jose, Toledo District, **Belize**	BzTDSJ04	TcIV*	ND
10	**Colombia**	CO1	TcI	ND
11	**Colombia**	CO6	TcI	ND
12	**Colombia**	CO8	TcI	TcI
13	Los Angeles, San Rafael, **Costa Rica**	CRHeSR01	TcI	TcI*
14	Santo Domingo, Heredia, **Costa Rica**	CRHeSD02	TcI	ND
15	Santo Domingo, Heredia, **Costa Rica**	CRHeSD04	TcI	ND
16	Santo Domingo, Heredia, **Costa Rica**	CRHeSD07	TcI	TcI*
17	Santo Domingo, Heredia, **Costa Rica**	CRHeSD11	TcI	ND
18	Santo Domingo, Heredia, **Costa Rica**	CRHeSD13	TcI	TcI*
19	Monte Largo, Santa Ana, **El Salvador**	ESSASA02Cl10	TcI	ND
20	Monte Largo, Santa Ana, **El Salvador**	ESSASA03	ND	TcI*
21	Monte Largo, Santa Ana, **El Salvador**	ESSASA12	TcI	ND
22	Monte Largo, Santa Ana, **El Salvador**	ESSASA21	ND	TcI*
23	**Guatemala**	GtQuSa01	TcI	ND
24	**Guatemala**	GtQuSa02	TcI	TcI
25	Lanquin, Alta Verapaz, **Guatemala**	GtAVLa06	TcI	TcI*
26	Lanquin, Alta Verapaz, **Guatemala**	GtAVLa07	TcI*	TcI*
27	Lanquin, Alta Verapaz, **Guatemala**	GtAVLa08	TcI*	ND
28	Lanquin, Alta Verapaz, **Guatemala**	GtAVLa02Cl12	TcI	ND
29	Jutiapa, **Guatemala**	GtJu01Cl24	TcI	ND
30	Jutiapa, **Guatemala**	GtJu02Cl26	TcI	ND
31	Jutiapa, **Guatemala**	GtJu03Cl31	TcI	ND
32	Jutiapa, **Guatemala**	GtJu04Cl36	TcI	ND
33	Jutiapa, **Guatemala**	GtJu05Cl41	TcI	ND
34	Jutiapa, **Guatemala**	GtJu06Cl52	TcI	ND
35	San Antonio, Copan, **Honduras**	HnCoSA05	TcI*	TcI*
36	San Antonio, Copan, **Honduras**	HnCoSA06	TcI*	TcI*
37	San Antonio, Copan, **Honduras**	HnCoSA17	ND	TcI*
38	San Antonio, Copan, **Honduras**	HnCoSA18	TcI	ND
39	San Marco de Sierra, Intibuca, **Honduras**	HnlnSM11	TcI	ND
40	San Marco de Sierra, Intibuca, **Honduras**	HnlnSM12	TcI	TcI*
41	San Marco de Sierra, Intibuca, **Honduras**	HnlnSM13	TcI*	ND
42	San Marco de Sierra, Intibuca, **Honduras**	HnlnSM14	ND	TcI*
43	Benito Juarez, Quintana Roo, **Mexico**	MxQRBJ03	TcI*	ND
44	Benito Juarez, Quintana Roo, **Mexico**	MxQRBJ04	TcIV	ND
45	Benito Juarez, Quintana Roo, **Mexico**	MxQRBJ05	ND	TcI
46	Calkini, Campeche, **Mexico**	MxCaCa05	TcI*	TcI*
47	Calkini, Campeche, **Mexico**	MxCaCa06	TcI*	TcI*
48	Calkini, Campeche, **Mexico**	MxCaCa07	TcI*	TcI*
49	Teya, Yucatan, **Mexico**	MxYuTe15	TcI*	ND
50	Yucatan, **Mexico**	MxYu01	ND	TcI
51	Yucatan, **Mexico**	MxYu02	ND	TcI

SL-IR = spliced leader intergenic region

ND = not determined,

* = single-stranded sequencing

### Genetic diversity of TcI strains

Diversity measures (S, h H_d_, π, and Tajima’s D) of 18S rDNA and SL-IR haplotypes were calculated in DNAsp (v. 5.10.01) [31].

### Tests for genetic structure among TcI isolates

To test for associations of TcI haplotypes among geographic regions, ecotopes and host/vector associations, haplotypes were analyzed by Principle Component Analysis (PCA) and Median-Joining Network analysis. These analyses used our sequences (Table 1) combined with those available on GenBank that were of sufficient length and had no ambiguous nucleotides (S2 and S3 Tables). The GenBank sequences were a mix of isolates, molecular clones, and cellular clones obtained originally from humans, wild mammals, and triatomine vectors. “Rodent” includes both *R*. *rattus* and other rodent species. The majority of the 18S TcI sequences were from Colombia and Brazil, with one each from Venezuela and Panama. SL-IR sequences were from Argentina, Brazil, Bolivia, Chile, Colombia, French Guyana, Mexico, Panama, Paraguay, Venezuela, and the USA. Since Colombia was so heavily sampled we chose to identify those sequences separately from the other broad geographic regions. Sequences were aligned in MacVector (v. 14.5.3, Apex, North Carolina) using Muscle [32]. The 18S TcI alignment contained 116 sequences including 27 sequences (this study), 87 from GenBank, and two outgroups: TcII (reference strain IVVcl4 [33], sequenced in our lab), and TcBat (JQ965548). The total 18S rDNA alignment including gaps was 179 bp, and excluding gaps sequences ranged from 155 to 178 base pairs. For SL-IR, only the single nucleotide polymorphism (SNP) region was used for network analysis because studies have shown that alignments of the microsatellite region are ambiguous [34, 35]. The alignment of the 185 SL-IR TcI sequences included 16 sequences (this study), 171 from GenBank, and one TcBat outgroup (TCC 203, KT305859.1) The total SL-IR alignment including gaps was 231 bp with sequences ranging from 221–223 bp, excluding gaps.

For the two markers, 18S and SL-IR, genetic differences among ecotopes and geographic regions were visualized using Principle Components Analysis in GenAlEx ver 6.502 [36] to plot genetic relationships among individuals and identify those from the same geographic region or ecotope. Individuals close to each other in the graph are more closely related to each other than to distant individuals. Nominal logistic regression was used to test for differences among groups based on the principle components (JMP Pro, Version 12.0. SAS Institute Inc., Cary, NC).

For the two markers, 18S and SL-IR, phylogenetic relationships among individuals were visualized using Median-Joining Networks and color-coded to identify those from the same geographic region, host/vector or ecotope. Median-Joining network analysis is preferred to phylogenetic inference for intraspecific analyses [37]. The sequences were analyzed using Network, DNA alignment and Network Publisher (fluxus-engineering.com, version 5.0.0.0). Median-Joining (MJ) Networks [38] were calculated and the post-processing maximum parsimony cleanup procedure [39] performed for both genes. Networks were then arranged by hand and nodes colored using Network Publisher.

In addition, isolation-by-distance was tested (GenAlEx ver. 6.5, [36, 40]) using the number of differences between sequences as a measure of distance.

## Results

### *T*. *cruzi* detection and strain determination

**Fig 1:**
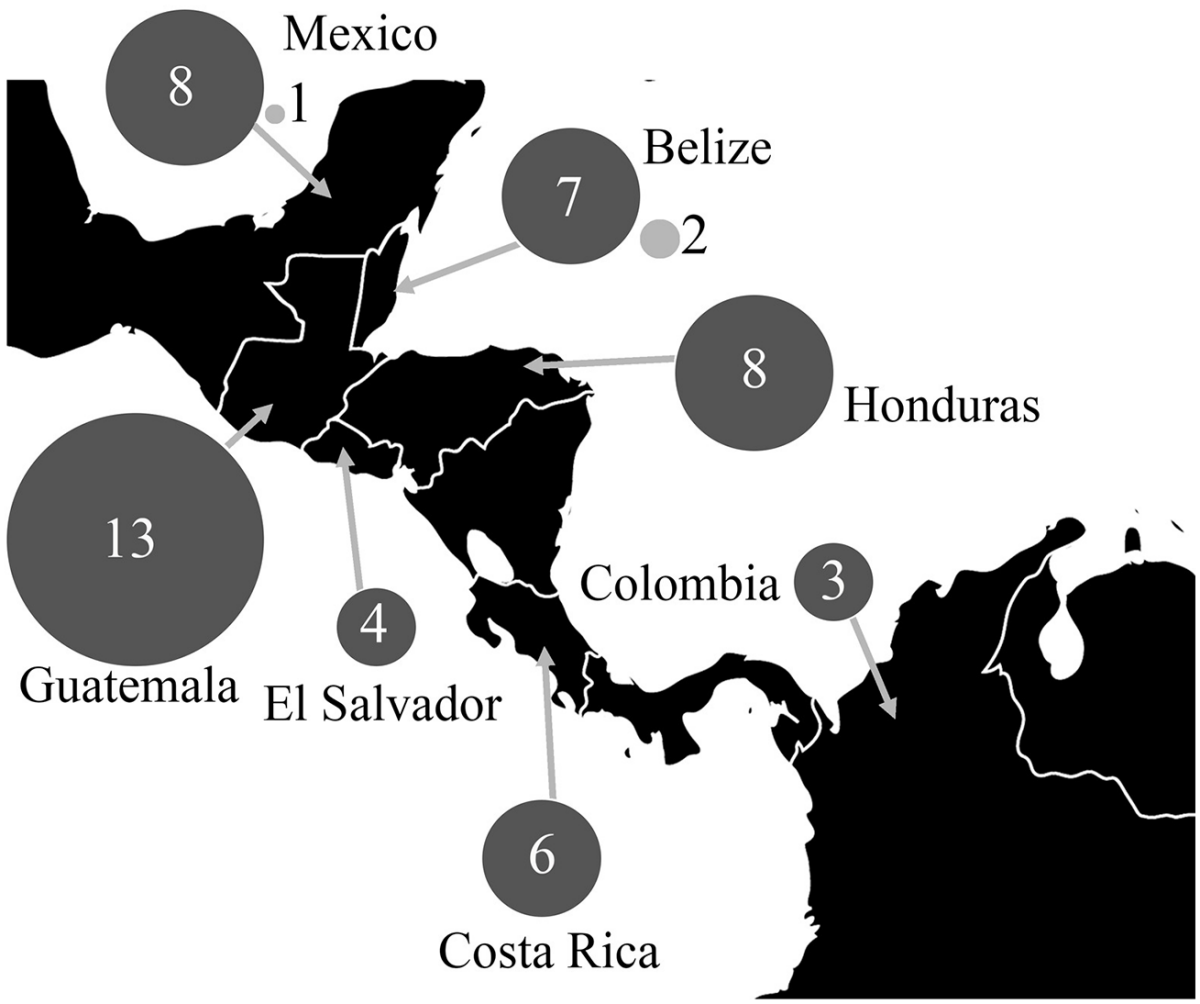
*Trypanosoma cruzi* strains TcI and TcIV identified in *Triatoma dimidiata* from Mexico, Central America and Colombia as determined by 18S rDNA and SL-IR (spliced leader intergenic region) sequences. Size of the circles is proportional to the numbers of *T*. *dimidiata* specimens with a particular *T*. *cruzi* strain (TcI -dark gray or TcIV—light gray) in different countries. Political map was modified from: https://commons.wikimedia.org/wiki/Atlas_of_the_world#/media/File:BlankMap-World6.svg under public domain.

TcI was the predominant DTU found in *T*. *dimidiata* based on the DNA sequence of two markers, 18S rDNA and the SL-IR. Thirty-eight percent (126/334) of the *T*. *dimidata* specimens examined were infected with *T*. *cruzi*, showing the *T*. *cruzi*-specific band by PCR. We were able to strain type 51 of the infected *T*. *dimidiata* and TcI was present in 94% (48) of these specimens based on ≥97% query coverage and ≥98% identity to *T*. *cruzi* specimens identified as TcI. Eighty-six percent (44/51) were determined based on 18S rDNA sequence, 43% (22/51) by the SL-IR sequence, and 29% (15/51) by both sequences (Table 1 and Fig 1, GenBank accession numbers: MF099414-MF099427). Where sequence was available from both markers, strain identifications were concordant. TcIV was found at a much lower prevalence, 6% (3/51) of the *T*. *dimidiata* tested, all determined by 18S rDNA sequence (Table 1 and Fig 1). The few TcIV identified were all found in the northern end of the range of *T*. *dimidiata*, in Yucatan, Mexico and Belize.

### Genetic diversity of TcI strains

Both genetic markers showed similar high haplotype diversity (nearly 1) and a low nucleotide diversity (2–3%), which suggested that almost every individual presents a unique haplotype and that haplotypes differ by few nucleotides (Table 2). The negative Tajima’s D suggested a bottleneck occurred in the recent past of the population’s evolution.

**Table 2:** Genetic Polymorphism and Diversity of TcI isolates.

Parameter	Genetic Marker	
	18S rDNA	SL-IR
N	114	184
Size (bp)	179	230
S	55	67
h	39	89
H_d_	0.952	0.921
π (Nucleotide Diversity)	0.020	0.027
Tajima’s D	-2.19	-2.11

N = number of sequences analyzed (Table 1 and S2 and S3 Tables) S = variable sites h = number of haplotypes H_d_ = haplotype diversity π = nucleotide diversity

### Genetic structure among TcI isolates

**Fig 2:**
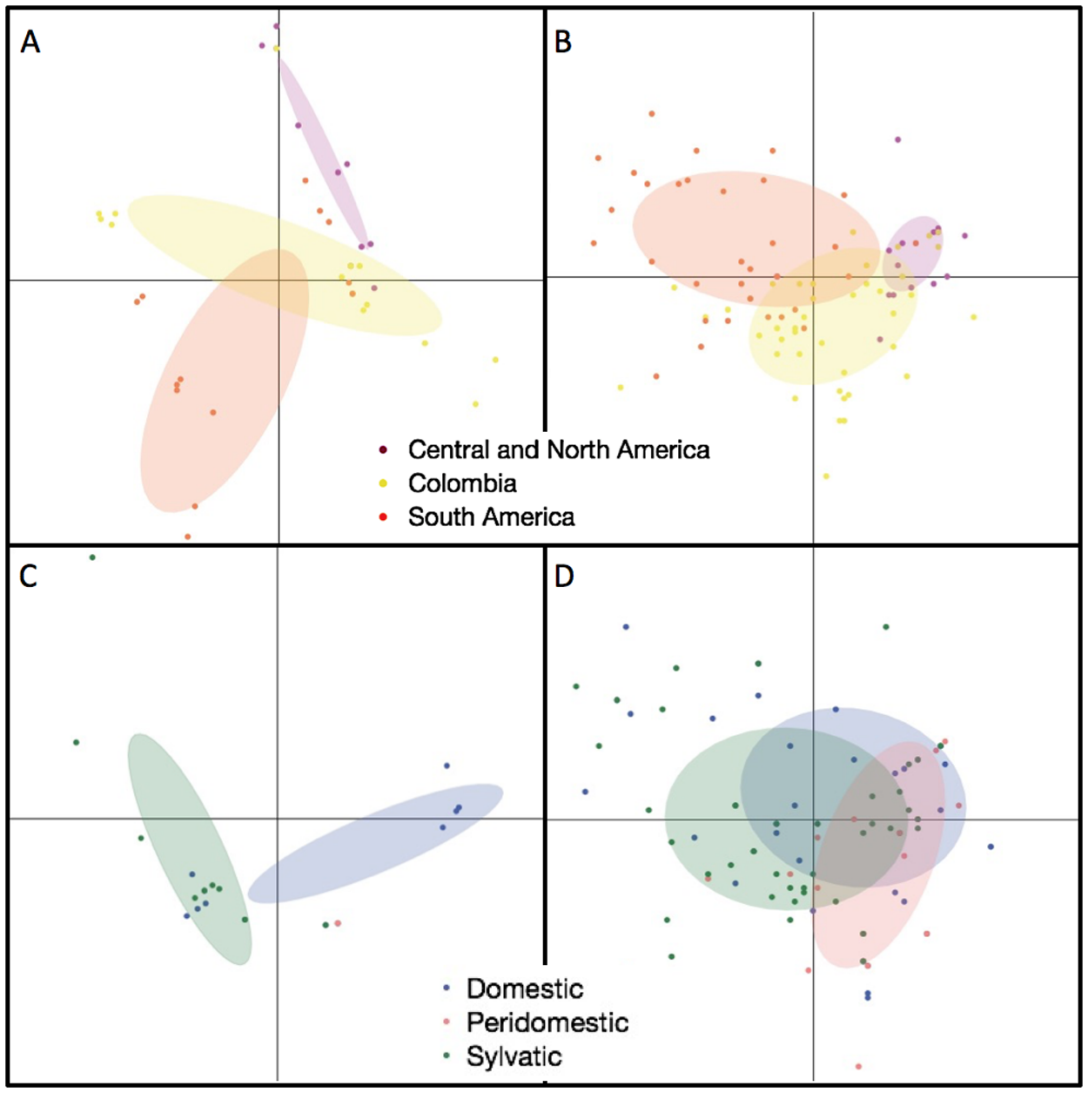
Principle component plots of variation in TcI isolates for the two markers by geographic region (A: 18S, B: SL-IR) and ecotope (C: 18S, D: SL-IR). Ellipses cover 50% of the variables. For geographic regions, both 18S and SL-IR are significantly different among regions. For ecotopes, the 18S sylvatic is significantly different from domestic, but for SL-IR, the differences are not statistically significant.

Significant differences in TcI haplotypes among geographic regions (North and Central America / Colombia / South America) were evident in the PCA for both markers, 18S and SL-IR (Fig 2A and 2B). The variation among geographic regions in both 18S and SL-IR was statistically significant (18S: Chi-Square 44.8, n = 109, d.f. = 2, < 0.0001; SL-IR: Chi-Square 133.8, n = 179, d.f. = 2, P < 0.0001). For the 18S marker the first two components explained 46.0% and 21.7%, or a total of 77.7% of the variance. For the SL-IR marker the first two components explained 31.9% and 19.4%, for a total of 51.3% of the variance (Fig 2A and 2B).

The PCA also showed significant differences in TcI haplotypes among ecotopes (sylvatic compared to domestic / peridomestic) for 18S but not SL-IR (Fig 2C and 2D). Logistic regression indicated that the 18S sylvatic haplotypes were significantly different from domestic and peridomestic (Chi-Square 38.3, n = 66, d.f. = 2, P < 0.0001), however, the haplotypes did not differ for the SL-IR marker (Chi-Square 5.0, n = 166, d.f. = 2, P > 0.05). For the 18S marker the first two components accounted for 61.6% and 16.6% of the variance for a cumulative total of 78.2% and for the SL-IR marker 31.8% and 20.4% of the variance for a total of 52.2% (Fig 2C and 2D).

### Network analysis

**Fig 3:**
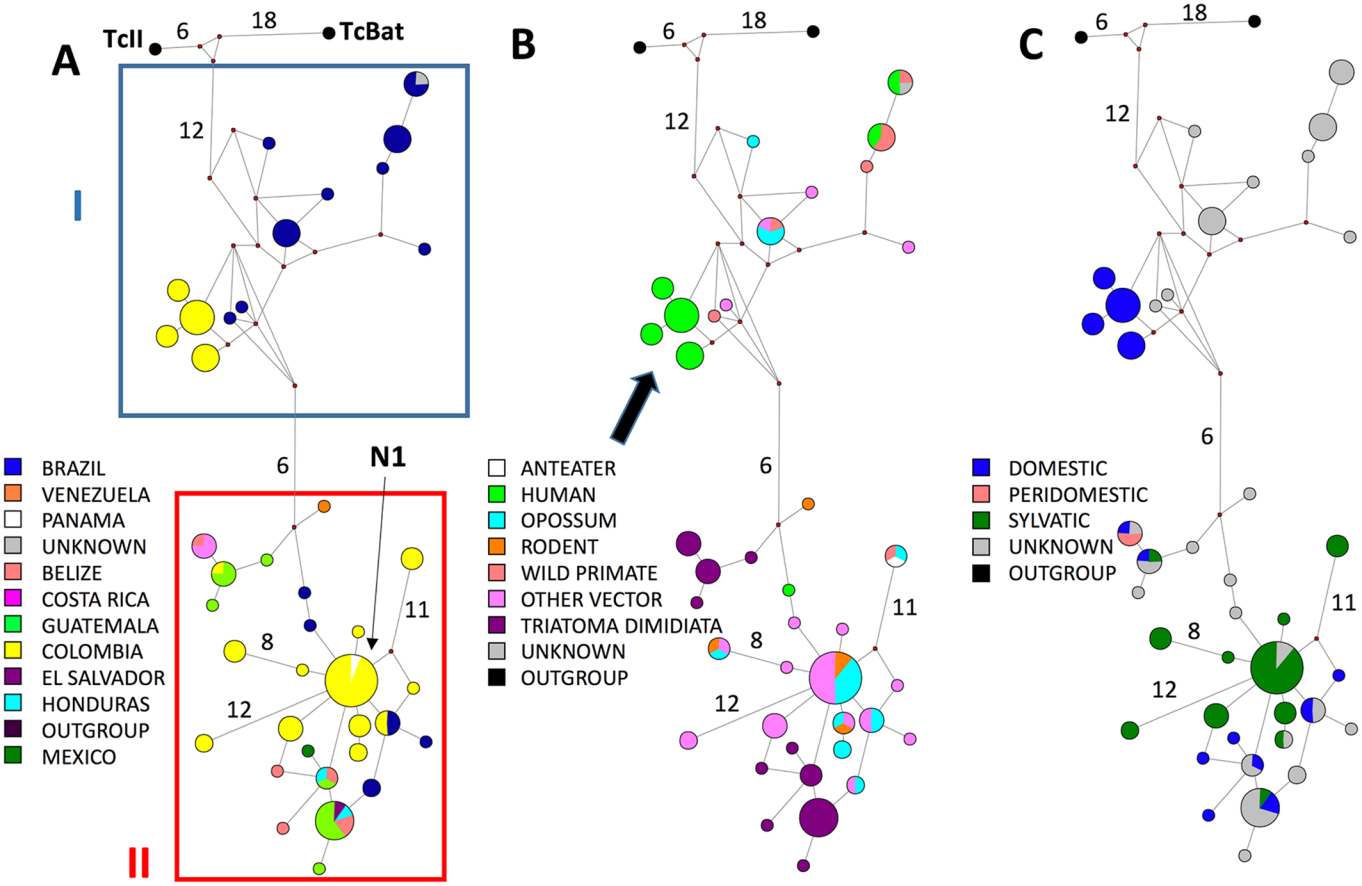
Median-Joining Network for 18S rDNA haplotypes. Networks were constructed with 39—18S haplotypes and the size of each node proportional to the frequency of the haplotype. Small red circles (median vectors) represent hypothetical intermediate nodes. TcII and TcBat are the outgroups. The number of mutational steps ≥3 are shown. Clustering is examined by: (A) geographic origin, (B) vector or host, and (C) ecotope.

The 18S MJ Network of TcI haplotypes included 39 haplotypes (10 from this study and 29 from GenBank), 49% (19/39) of the haplotypes contained only one sequence and one predominant haplotype contained 18 sequences (N1, Fig 3A). Geographic association of haplotypes was also evident in the MJ Network analysis where the majority of the Brazilian TcI haplotypes appeared in group I. The only other location represented in group I was a tight cluster of four Colombian haplotypes; only one mutational step separated each of these four haplotypes and represented 19 sequences. Group II contained all of the North and Central American haplotypes and the remaining Colombian haplotypes including the predominant haplotype (N1).

In general, haplotypes appeared to be spread across hosts and vectors by MJ Network analysis with the 18S marker (Fig 3B). For example, N1 contained TcI isolates from five different taxa. However, some haplotypes were only identified in *T*. *dimidiata* (two clusters within group II) and the cluster of four haplotypes from Colombia were all from humans (arrow, Fig 3B). Also by the 18S marker, TcI haplotypes from different ecotopes appeared to be spread across the network as was evident in Group 2 (Fig 3C). Ecotope data was mostly lacking for specimens in Group 1 with the exception of the cluster containing domestic isolates from humans in Colombia (Fig 3C).

**Fig 4:**
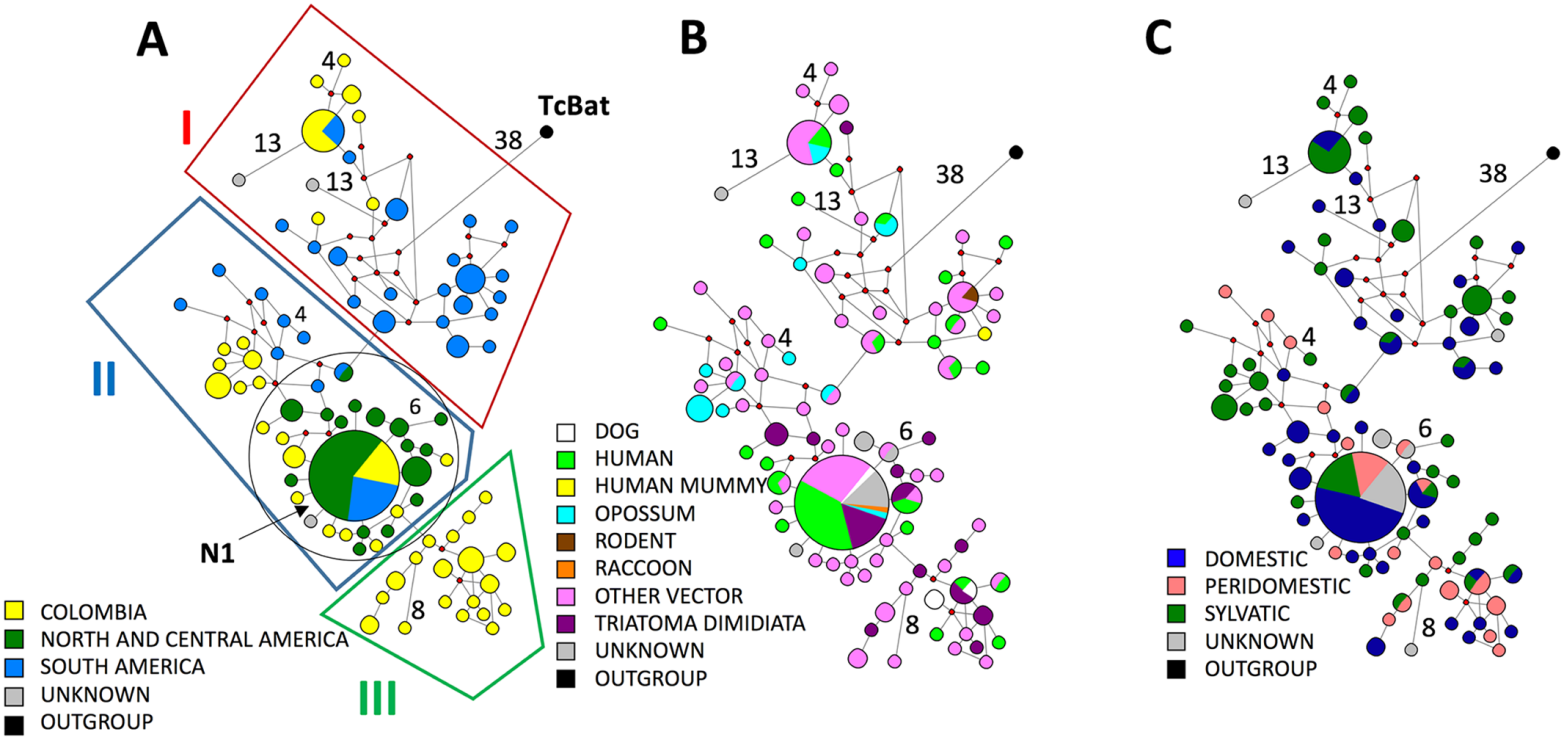
Median-Joining Network for the single nucleotide polymorphism region of the intergenic region of the spliced leader gene (SL-IR). Networks were constructed with 89—SL-IR haplotypes and the size of each node proportional to the frequency of the haplotype. Small red circles (median vectors) represent hypothetical intermediate nodes. TcBat is the outgroup. The number of mutational steps ≥3 are shown. Clustering is examined by: (A) geographic origin, (B) vector or host, and (C) ecotope.

The MJ SL-IR network included 89 haplotypes (four new from this study and 85 from GenBank), with 74% (66/89) of the nodes represented by a single sequence and one predominant haplotype containing 50 sequences (N1, Fig 4A); the remaining nodes contained between one and five sequences. Because of the large number of SL-IR sequences, we grouped the data into geographic regions instead of individual countries with the exception of Colombia because of the extensive sampling in this country. The predominant haplotype, N1, contained sequences from all three geographic regions (Fig 4A) and included 63% (10/16) of all countries represented in the data (Table 1 and S3 Table). Clustered close to N1 were nearly all the remaining TcI haplotypes from North and Central America (circle within Group II). Two branches extended off this cluster: one of just Colombian haplotypes (Group III) and one of South American and Colombian haplotypes (Group I). This SL-IR network showed no association between haplotype and host; indeed, N1 included isolates from 75% (9/12) of vector/host taxa (Fig 4B). In addition, there was no clear clustering of haplotypes and ecotopes. N1 haplotypes were from all ecotopes and haplotypes from sylvan and domestic ecotopes appeared to be spread throughout the network, although peridomestic haplotypes were lacking in group 1 (Fig 4C).

### Mantel test

Genetic distance was significantly correlated with geographic distance for both markers (S1 Fig) thus supporting an isolation-by-distance mechanism of genetic differentiation.

## Discussion

### *T*. *cruzi* strains present in *T*. *dimidiata*

This study shows that TcI is the predominant DTU in *T*. *dimidiata* collected across its geographic range from Mexico, through Central America and into Colombia. Indeed, ninety-four percent of the *T*. *cruzi*-infected *T*. *dimidiata* contained TcI (Table 1, Fig 1). TcIV was the only other DTU found, at a much lower frequency, 6% of the *T*. *cruzi* positive *T*. *dimidiata*. As previously 90.7% of *T*. *cruzi* DTUs reported were from South America [20], this study provides important information about strains circulating in Central America and Mexico and in the most important vector in this region, *T*. *dimidiata*. Furthermore, because only a subset of the strains known from South America (2/7) were found in Central America and Mexico, these results do not challenge the South American origin hypothesis for *T*. *cruzi* [41]; a broader sampling will be required to answer this question.

Our results are in accordance with other studies that show that TcI is the predominant DTU across Latin America and more specifically in the geographic range of *T*. *dimidiata*: Mexico [12, 17, 42], Central America [14, 18, 23, 24], and northern South America [11, 16]. TcIV is also the most commonly reported secondary strain in this region and the ratio we found (94% TcI / 6% TcIV) is nearly identical to previous reports from Central America (93.3% TcI / 6.7% TcIV [20]). We did not find any of the rarely reported other strains in our *T*. *dimidiata* specimens [26, 27, 42]. This may be because our study did not include specimens from central Mexico or the southern Yucatan peninsula where these strains were identified. In addition, the infection prevalence we observed (38%) is quite comparable to what was previously reported in Guatemalan *T*. *dimidiata* (39%), also determined by PCR [43]. The predominance of TcI and the high prevalence in *T*. *dimidiata* (nearly 40% are carrying the parasite) mean that this strain, in the most important vector in Central America, is responsible for the majority of Chagas disease in this region. A broader sampling of *T*. *cruzi* strains in other hosts and vectors will better clarify the epidemiology of Chagas in this region.

TcI is also genetically quite diverse across the geographic range of *T*. *dimidiata*. A high diversity across this continental scale was also observed by Llewellyn, et al. [21]. The high diversity found in Central American TcI isolates, if it holds with additional sampling, may challenge the South American origin hypothesis of TcI [21, 35]. Moreover, the clustering the majority of the Central/North American TcI isolates in and around the predominant node in the MJ Networks (Figs 3 & 4A) suggests that Central/North America may actually be the origin of TcI. A broader sampling, especially of other vectors and hosts, is needed to resolve this question. Importantly, we amplified directly from *T*. *dimidiata* abdominal DNA, therefore avoiding the biases resulting from culturing isolates prior to sequencing [44].

We found evidence of geographic structuring of TcI haplotypes by two markers and three types of analysis. First, significant separation of haplotypes between North and Central America / Colombia / South America was shown by PCA using both markers, 18S and SL-IR (Fig 2A and 2B). Second, MJ Network analysis with both markers shows geographic separation of TcI haplotypes between South America and North/Central America, with the exception of Colombian haplotypes, which were found in both groups (Figs 3A and 4A). Third, a significant correlation between genetic and geographic distance by the Mantel test also supports geographic structure, suggesting isolation by distance. This result is consistent with other studies that also found genetic and geographic structure in TcI isolates [19, 21, 35].

Although there is strong support for geographic structuring among TcI isolates, there is weak support for structure among ecotopes. By PCA, only one marker (18S) showed statistically significant differences between domestic/peridomestic and sylvan isolates (Fig 2C and 2D). This significant difference could just reflect geographic structuring as ecotope information is largely absent for isolates from South America from which 18S sequence was determined, with the exception of the domestic cluster from humans in Colombia. The difference in results between the two markers may also reflect sampling of different *T*. *cruzi* populations: 18S sequences are nearly all from specimens from Colombia and Brazil, whereas SL-IR sequences are from a broader geographic range. Ecotope structuring is also not supported by MJ Network for the SL-IR marker. There is also no evidence of host/vector association by MJ networks with either marker, consistent with previous studies [35, 45]. A notable exception is an interesting human cluster from Colombia, evident in the 18S MJ network (Fig 3B, arrow).

The high similarity between these human TcI isolates from Colombia is not due to geographic proximity. In fact, the four nodes include 19 sequences that originate from geographically distant localities within Columbia, including six departments. It is possible that these represent the TcI subgroup, TcI_DOM_. TcI_DOM_ has been described using the SL—IR [19, 21, 22], and later also identified using *cyt* b [22]. However, this TcI_DOM_ subgroup has not previously been typed using the 18S marker, and in our SL—IR network the subgroup was not observed, limiting the ability to correlate with the previously published TcI_DOM._ Suggesting that it is a distinct subgroup is the observation that the subgroup we identified clusters with the South American isolates, not the North/Central American isolates as was reported for TcI_DOM_. It will be important to check these isolates with multiple markers to confirm or refute an association with TcI_DOM._

This study provides new information about *T*. *cruzi* strains circulating within *T*. *dimidiata* across its large geographic range. Our results indicate that TcI predominates from Mexico through Central America, extending into Colombia and TcIV is also present in *T*. *dimidiata* collected from Mexico and Belize. Central American TcI strains add to the tremendous diversity found within TcI and provide additional evidence for geographic structuring, and a lack of evidence of host/vector or strong ecotope association. The high diversity found within this *T*. *cruzi* strain may challenge vaccine development and treatment improvement, if the genetically different strains respond differently to particular medications. The lack of host/vector and ecotope association suggests the parasite (via the vector) is moving frequently between hosts and ecotopes. These results support previous studies showing that *T*. *dimidiata* is a quite mobile vector [46] so that reinfestation some months following pesticide treatment is common [47]. This lends further support to development approaches for Chagas control in Central America/southern Mexico such as the Ecohealth approach [48, 49], which uses local materials and community participation to improve houses. Unlike the temporary effects of pesticide application, the Ecohealth approach makes houses refractory to the vectors long-term, so is likely to be more effective for sustainable interruption of transmission.

## Supporting information

S1 Table Complete list of *Triatoma dimidiata* specimens, collecting and *Trypanosoma cruzi* infection information. Blank lines, except for first column, indicate data is not available.
(PDF)

S2 Table GenBank 18S rDNA sequences from TcI isolates used in study. (PDF)

S3 Table GenBank Spliced Leader—Intergenic Region (SL-IR) sequences from TcI isolates used in study. (PDF)

S1 Fig Mantel Test showing significant correlation with genetic and geographic distance for: (A) 18S rDNA and (B) SL-IR sequences. (TIF)
