# Supplementary material for: The diversity of the Chagas parasite, Trypanosoma cruzi, infecting the main Central American vector, Triatoma dimidiata, from Mexico to Colombia
Source: PLoS Negl Trop Dis. 2017 Sep 28;11(9):e0005878. doi: 10.1371/journal.pntd.0005878 (PMC5619707; doi:10.1371/journal.pntd.0005878)
Supplement: S1 Table — Blank lines, except for first column, indicate data is not available. (PDF) [file pntd.0005878.s001.pdf]

Specimen ID: Specimens Used in Strain-typing	Specimen Lab ID	Sex	Ecotope, D = domestic, P = peridomestic, S = sylvan	Country	Latitude	Longitude	*T. cruzi* Infection
	A1669	Female		Guatemala	15.288	-91.0883	negative
	A1670			Guatemala	15.288	-91.0883	negative
	A2272	Female		Guatemala	14.188	-90.2775	negative
	A2560			Guatemala	15.288	-91.0883	negative
	A2593			Guatemala	15.288	-91.0883	negative
	A2594			Guatemala	15.288	-91.0883	positive
	A2684			Guatemala	15.288	-91.0883	negative
	A2685			Guatemala	15.288	-91.0883	negative
	A2686			Guatemala	15.288	-91.0883	negative
	A2687	Female		Guatemala	15.288	-91.0883	negative
	A2688		P	Guatemala	15.288	-91.0883	positive
	A2689			Guatemala	15.288	-91.0883	negative
	A2730			Guatemala	15.288	-91.0883	positive
	A2731	Female	S	Guatemala	17.057	-89.9917	positive
	A2859	Female	S	Guatemala	17.057	-89.9917	positive
GtAVLa02	A2861	Male	S	Guatemala	17.057	-89.9917	positive
GtAVLa07	A2907	Female	S	Guatemala	17.057	-89.9917	positive
	A2952	Female		Guatemala	15.175	-90.9433	positive
	A2953	Male		Guatemala	15.175	-90.9433	positive
	A2997	Male		Guatemala	14.111	-89.9144	negative
	A3001	Male		Guatemala	14.111	-89.9144	positive
GtQuSa01	A3003			Guatemala	15.288	-91.0883	positive
GtQuSa02	A3024		P	Guatemala	15.288	-91.0883	positive
GtAVLa08	A3129	Male	S	Guatemala	17.057	-89.9917	positive
GtAVLa06	A3570	Female	S	Guatemala	17.057	-89.9917	positive
	A3571	Male	S	Guatemala	17.057	-89.9917	negative
	A3572	Male	S	Guatemala	17.057	-89.9917	negative
	A3573	Male	S	Guatemala	17.057	-89.9917	negative
	A3881	Female	S	Guatemala	17.057	-89.9917	negative
	A4530	Male		Guatemala	14.262	-90.1294	positive
	A4534	Male		Guatemala	14.262	-90.1294	negative
	A4538	Female		Guatemala	14.262	-90.1294	negative
	A4550	Male		Guatemala	14.262	-90.1294	negative
	A4606	Male		Guatemala	14.262	-90.1294	negative
	A4610	Male		Guatemala	14.262	-90.1294	negative
	A4625	Male		Guatemala	14.262	-90.1294	positive
GtJu01	A4631	Male		Guatemala	14.262	-90.1294	positive
	A4632	Male		Guatemala	14.262	-90.1294	negative
GtJu02	A4633	Male		Guatemala	14.262	-90.1294	positive
GtJu03	A4634	Male		Guatemala	14.262	-90.1294	positive
	A4645	Male		Guatemala	14.262	-90.1294	negative
GtJu04	A4666	Male		Guatemala	14.262	-90.1294	positive
	A4672	Male		Guatemala	14.262	-90.1294	negative
GtJu05	A4673	Male		Guatemala	14.262	-90.1294	positive
GtJu06	A4676	Male		Guatemala	14.262	-90.1294	positive
	A5096	Male		Guatemala	14.111	-89.9144	positive
	A5098	Male		Guatemala	14.111	-89.9144	negative
	A5099	Male		Guatemala	14.111	-89.9144	negative
	A5101	Male		Guatemala	14.111	-89.9144	negative
	A5158	Female		Guatemala			positive
	A5200	Female		Guatemala	15.175	-90.9433	negative
	A5201	Male		Guatemala	15.175	-90.9433	negative

Specimen ID: Specimens Used in Strain-typing	Specimen Lab ID	Sex	Ecotope, D = domestic, P = peridomestic, S = sylvan	Country	Latitude	Longitude	*T. cruzi* Infection
	A5260	Female		Guatemala	14.111	-89.9144	negative
	A5261	Male		Guatemala	14.111	-89.9144	positive
	A5448	Male		Guatemala	14.111	-89.9144	positive
	A5450	Female		Guatemala	14.111	-89.9144	positive
	A5617	Male		Guatemala	14.111	-89.9144	positive
	A5623	Female		Guatemala	14.111	-89.9144	negative
	A5700			Guatemala			positive
	A5797			Guatemala			negative
	A5659	Female	S	Guatemala	17.057	-89.9917	negative
	A5948	Male		Guatemala	15.175	-90.9433	negative
	A5949	Female		Guatemala	15.175	-90.9433	negative
	A6048	Female	S	Guatemala			negative
	A6051	Male	S	Guatemala	17.057	-89.9917	positive
	A6052	Female	S	Guatemala	17.057	-89.9917	negative
	GD0325	Female		Guatemala	17.247	-90.2928	negative
	GD0326	Female		Guatemala	17.247	-90.2928	positive
	GD327	Male		Guatemala	17.247	-90.2928	negative
CO1	co-1	Male	D	Colombia			positive
	co-2	Male	D	Colombia			negative
	co-3	Male	D	Colombia			negative
	co-4	Male	D	Colombia			negative
	co-5	Male	D	Colombia			negative
CO6	co-6	Male	D	Colombia			positive
	co-7	Male	D	Colombia			negative
CO8	co-8	Male	D	Colombia			positive
	co-9	Male	D	Colombia			negative
	co-10	Male	D	Colombia			positive
	co-11	Male	D	Colombia			positive
	co-12	Male	D	Colombia			negative
	co-13	Male	D	Colombia			positive
	Ecuador 1	Female	D	Ecuador			positive
	Ecuador 2	Male	D	Ecuador			negative
	Ecuador 3	Male	D	Ecuador			positive
	Ecuador 4	Male	D	Ecuador			positive
	Ecuador 5	Female	D	Ecuador			negative
	Ecuador 6	Male	D	Ecuador			positive
	Ecuador 7	Female	D	Ecuador			positive
	Ecuador 8	Male	D	Ecuador			negative
	Ecuador 9	Male	D	Ecuador			negative
	Ecuador 10	Male	D	Ecuador			negative
	Ecuador 11	Male	D	Ecuador			positive
	Ecuador 12	Male	D	Ecuador			negative
	Ecuador 13	Male	D	Ecuador			negative
	Ecuador 14		D	Ecuador			negative
	Ecuador 15		D	Ecuador			negative
	Ecuador 16		D	Ecuador			negative
CRHeSR01	17aCR	Male		Costa Rica			positive
	20aCR	Male	P	Costa Rica	10.039	-84.0908	positive
	22aCR	Male	P	Costa Rica	10.039	-84.0908	negative
	28aCR	Male	P	Costa Rica	10.039	-84.0908	positive
	30aCR	Male	P	Costa Rica	10.039	-84.0908	negative
	31aCR	Male	P	Costa Rica	10.039	-84.0908	positive

Specimen ID: Specimens Used in Strain-typing	Specimen Lab ID	Sex	Ecotope, D = domestic, P = peridomestic, S = sylvan	Country	Latitude	Longitude	*T. cruzi* Infection
	34aCR	Male	P	Costa Rica	10.039	-84.0908	negative
	35aCR	Male	P	Costa Rica	10.039	-84.0908	positive
	39aCR	Male	P	Costa Rica	10.039	-84.0908	negative
	40aCR	Female	P	Costa Rica	10.039	-84.0908	negative
	42aCR	Female	P	Costa Rica	10.039	-84.0908	negative
	43aCR	Female	P	Costa Rica	10.039	-84.0908	negative
	45aCR	Female	P	Costa Rica	10.039	-84.0908	positive
	46aCR	Female	P	Costa Rica	10.039	-84.0908	positive
	47aCR	Female	P	Costa Rica	10.039	-84.0908	negative
	48aCR	Female	P	Costa Rica	10.039	-84.0908	negative
	52aCR	Male	P	Costa Rica	10.039	-84.0908	positive
CRHeSD02	57aCR	Female	P	Costa Rica	9.9958	-84.0731	positive
	58aCR	Female	P	Costa Rica	9.9958	-84.0731	negative
CRHeSD04	59aCR	Female	P	Costa Rica	9.9958	-84.0731	positive
	60aCR	Female	P	Costa Rica	9.9958	-84.0731	positive
	61aCR	Female	P	Costa Rica	9.9958	-84.0731	positive
CRHeSD07	64aCR	Female	P	Costa Rica	9.9958	-84.0731	positive
	65aCR	Female	P	Costa Rica	9.9958	-84.0731	positive
	68aCR	Male	P	Costa Rica	9.9958	-84.0731	negative
	70aCR	Male	P	Costa Rica	9.9958	-84.0731	positive
CRHeSD11	71aCR	Male	P	Costa Rica	9.9958	-84.0731	positive
	74aCR	Male	P	Costa Rica	9.9958	-84.0731	positive
CRHeSD13	80aCR	Male	P	Costa Rica	9.9958	-84.0731	positive
	82aCR	Male	P	Costa Rica	9.9958	-84.0731	positive
HnCoSA17	HN05 01/97	Female		Honduras	15.033	-88.8833	positive
HnCoSA05	HN05 01/96	Female		Honduras	15.033	-88.8833	positive
	HN05 01/92	Female		Honduras	15.033	-88.8833	negative
	HN05 03/108	Female		Honduras	15.033	-88.8833	negative
	HN05 04/118	Female		Honduras	15.033	-88.8833	negative
	HN05 02/105	Female		Honduras	15.033	-88.8833	negative
	HN05 01/91	Female		Honduras	15.033	-88.8833	negative
	HN05 03/112	Male		Honduras	15.033	-88.8833	negative
	HN05 03/113	Male		Honduras	15.033	-88.8833	negative
	HN05 04/115	Male		Honduras	15.033	-88.8833	negative
	HN05 02/99	Male		Honduras	15.033	-88.8833	positive
	HN05 02/103	Male		Honduras	15.033	-88.8833	positive
	HN05 01/93	Male		Honduras	15.033	-88.8833	negative
	HN05 02/101	Male		Honduras	15.033	-88.8833	negative
	HN04 01/58	Female		Honduras	14.117	-88.233	negative
	HN04 02/69	Female		Honduras	14.117	-88.233	negative
	HN04 01/57	Female		Honduras	14.117	-88.233	negative
HnInSM14	HN04 02/70	Female		Honduras	14.117	-88.233	positive
HnInSM11	HN04 03/76	Female		Honduras	14.117	-88.233	positive
	HN04 04/83	Female		Honduras	14.117	-88.233	negative
	HN04 04/82	Female		Honduras	14.117	-88.233	positive
HnInSM13	HN04 03/81	Female		Honduras	14.117	-88.233	positive
	HN04 04/88	Female		Honduras	14.117	-88.233	negative
	HN04 02/68	Female		Honduras	14.117	-88.233	negative
	HN04 01/61	Male		Honduras	14.117	-88.233	negative
	HN04 03/71	Male		Honduras	14.117	-88.233	positive
	HN04 02/64	Male		Honduras	14.117	-88.233	negative
	HN04 02/66	Male		Honduras	14.117	-88.233	negative

Specimen ID: Specimens Used in Strain-typing	Specimen Lab ID	Sex	Ecotope, D = domestic, P = peridomestic, S = sylvan	Country	Latitude	Longitude	*T. cruzi* Infection
	HN04 02/65	Male		Honduras	14.117	-88.233	negative
	HN04 03/75	Male		Honduras	14.117	-88.233	negative
	HN04 01/62	Male		Honduras	14.117	-88.233	negative
	HN04 02/67	Male		Honduras	14.117	-88.233	negative
	HN04 03/72	Male		Honduras	14.117	-88.233	positive
	HN04 01/60	Male		Honduras	14.117	-88.233	negative
	HN04 02/63	Male		Honduras	14.117	-88.233	positive
HnInSM12	HN04 03/80	Male		Honduras	14.117	-88.233	positive
	HN04 04/85	Male		Honduras	14.117	-88.233	negative
	HN04 03/74	Male		Honduras	14.117	-88.233	negative
	HN04 04/86	Male		Honduras	14.117	-88.233	negative
	HN05 01/89	Female		Honduras	15.033	-88.8833	negative
	HN05 03/107	Female		Honduras	15.033	-88.8833	negative
HnCoSA06	HN05 01/98	Female		Honduras	15.033	-88.8833	positive
	HN05 04/117	Female		Honduras	15.033	-88.8833	negative
	HN05 03/111	Male		Honduras	15.033	-88.8833	negative
	HN05 01/95	Male		Honduras	15.033	-88.8833	negative
HnCoSA18	HN05 02/102	Male		Honduras	15.033	-88.8833	positive
	HN05 04/116	Male		Honduras	15.033	-88.8833	negative
	HN05 03/104			Honduras			negative
	HN05 03/106			Honduras			negative
	HN05 03/110	Male		Honduras	15.033	-88.8833	negative
	HN05 04/114	Male		Honduras	15.033	-88.8833	negative
	HN05 02/100	Male		Honduras	15.033	-88.8833	negative
	HN05 01/94	Male		Honduras	15.033	-88.8833	negative
	s128	Female	D	El Salvador	13.979	-89.6142	positive
ESSASA02Cl10	s130	Female	D	El Salvador	13.979	-89.6142	positive
ESSASA03	s131	Male	D	El Salvador	13.979	-89.6142	positive
	s132	Female	D	El Salvador	13.979	-89.6142	negative
	s133	Male	D	El Salvador	13.979	-89.6142	positive
	s134	Male	D	El Salvador	13.979	-89.6142	negative
	s135	Female	D	El Salvador	13.979	-89.6142	negative
	s136	Male	D	El Salvador	13.979	-89.6142	positive
	s137	Female	D	El Salvador	13.979	-89.6142	negative
	s138	Female	D	El Salvador	13.979	-89.6142	negative
	s139	Female	D	El Salvador	13.979	-89.6142	positive
	s140	Male	D	El Salvador	13.979	-89.6142	positive
	s141	Female	D	El Salvador	13.979	-89.6142	positive
ESSASA21	s142	Female	D	El Salvador	13.979	-89.6142	positive
ESSASA12	s143	Female	D	El Salvador	13.979	-89.6142	positive
	s144	Male	D	El Salvador	13.979	-89.6142	negative
	s145	Female	D	El Salvador	13.979	-89.6142	negative
	s146	Female	D	El Salvador	13.979	-89.6142	positive
	s147	Female	D	El Salvador	13.979	-89.6142	negative
	s148	Male	D	El Salvador	13.979	-89.6142	negative
	s149	Male	D	El Salvador	13.979	-89.6142	positive
	s150	Male	D	El Salvador	13.979	-89.6142	negative
	s151	Male	D	El Salvador	13.979	-89.6142	negative
	s155	Male	D	El Salvador	13.979	-89.6142	negative
	s156	Male	D	El Salvador	13.979	-89.6142	negative
	s161	Male	D	El Salvador	13.979	-89.6142	negative
	s162	Male	D	El Salvador	13.979	-89.6142	negative

Specimen ID: Specimens Used in Strain-typing	Specimen Lab ID	Sex	Ecotope, D = domestic, P = peridomestic, S = sylvan	Country	Latitude	Longitude	*T. cruzi* Infection
	yuc1658	Female		Mexico	20.374	-90.0522	negative
	yuc1666	Female		Mexico	20.374	-90.0522	negative
	yuc1669	Male		Mexico	20.374	-90.0522	negative
	yuc1671	Female		Mexico	20.374	-90.0522	negative
	yuc1672	Female		Mexico	20.374	-90.0522	negative
	yuc1673	Male		Mexico	20.374	-90.0522	negative
	yuc1674	Male		Mexico	20.374	-90.0522	negative
	yuc1675	Male		Mexico	20.374	-90.0522	negative
	yuc1676	Female		Mexico	20.374	-90.0522	negative
	yuc1680	Female	D	Mexico	20.374	-90.0522	positive
	yuc2131	Male		Mexico	20.374	-90.0522	negative
	yuc2132	Male		Mexico	20.374	-90.0522	negative
	yuc2143	Male	D	Mexico	20.374	-90.0522	positive
	yuc2144	Female		Mexico	20.374	-90.0522	negative
MxCaCa05	yuc2145	Male	D	Mexico	20.374	-90.0522	positive
	yuc2146	Male		Mexico	20.374	-90.0522	negative
MxCaCa06	yuc2504	Male	D	Mexico	20.374	-90.0522	positive
MxCaCa07	yuc2507	Male	D	Mexico	20.374	-90.0522	positive
	yuc2516	Male	D	Mexico	20.374	-90.0522	positive
	yuc2517	Male		Mexico	20.374	-90.0522	negative
	yuc2518	Female		Mexico	20.374	-90.0522	negative
	yuc2519	Female		Mexico	20.374	-90.0522	negative
	yuc2599	Female		Mexico	20.902	-87.4856	negative
	yuc2600	Female		Mexico	20.902	-87.4856	negative
	yuc2602	Female		Mexico	20.902	-87.4856	negative
	yuc2603-1			Mexico	20.902	-87.4856	negative
	yuc2603-2			Mexico	20.902	-87.4856	negative
MxQRBJ03	yuc2606	Male	D	Mexico	20.902	-87.4856	positive
	yuc2607	Male		Mexico	20.902	-87.4856	negative
	yuc2608	Male		Mexico	20.902	-87.4856	negative
	yuc2609	Male		Mexico	20.902	-87.4856	negative
MxQRBJ04	yuc2610	Female	D	Mexico	20.902	-87.4856	positive
	yuc2611	Female		Mexico	20.902	-87.4856	negative
	yuc2612	Female		Mexico	20.902	-87.4856	negative
MxQRBJ05	yuc2614	Male	D	Mexico	20.902	-87.4856	positive
MxYu01	yuc2615		D	Mexico			positive
	yuc2616			Mexico			negative
MxYu02	yuc2617		D	Mexico			positive
	yuc2618	Male		Mexico	20.902	-87.4856	negative
	yuc2619	Male		Mexico	20.902	-87.4856	negative
MxQRTR01	yuc2625	Female	D	Mexico	20.902	-87.4856	positive
	yuc2626	Female		Mexico	20.902	-87.4856	negative
	yuc2627	Female		Mexico	20.902	-87.4856	negative
	yuc271	Male		Mexico	20.902	-87.4856	negative
	yuc273	Male		Mexico	20.902	-87.4856	negative
	yuc277	Female		Mexico	20.902	-87.4856	negative
	yuc278	Female		Mexico	20.902	-87.4856	negative
	yuc293	Female		Mexico	20.902	-87.4856	negative
	yuc296	Male		Mexico	20.902	-87.4856	negative
	yuc3058	Female		Mexico	20.374	-90.0522	negative
	yuc3059	Female		Mexico	20.374	-90.0522	negative
	yuc3060	Female	D	Mexico	20.374	-90.0522	positive

Specimen ID: Specimens Used in Strain-typing	Specimen Lab ID	Sex	Ecotope, D = domestic, P = peridomestic, S = sylvan	Country	Latitude	Longitude	*T. cruzi* Infection
	yuc3061	Female		Mexico	20.374	-90.0522	negative
	yuc3073	Male		Mexico	20.374	-90.0522	negative
	yuc4329	Male	D	Mexico	21.051	-89.072	negative
	yuc4541	Male	D	Mexico	21.051	-89.072	negative
	yuc4591	Male	D	Mexico	21.051	-89.072	negative
	yuc4618	Male	D	Mexico	21.051	-89.072	negative
	yuc4621	Male	D	Mexico	21.051	-89.072	negative
	yuc4622	Male	D	Mexico	21.051	-89.072	negative
	yuc4624	Male	D	Mexico	21.051	-89.072	negative
	yuc4642	Male	D	Mexico	21.051	-89.072	negative
	yuc4654	Male	D	Mexico	21.051	-89.072	negative
	yuc4669	Male	D	Mexico	21.051	-89.072	negative
	yuc4670	Female	D	Mexico	21.051	-89.072	positive
	yuc4671	Female	D	Mexico	21.051	-89.072	positive
	yuc4675	Male	D	Mexico	21.051	-89.072	negative
	yuc4676	Male	D	Mexico	21.051	-89.072	negative
	yuc4677	Female	D	Mexico	21.051	-89.072	negative
	yuc4678	Female	D	Mexico	21.051	-89.072	positive
	yuc4679	Female	D	Mexico	21.051	-89.072	negative
	yuc4684	Female	D	Mexico	21.051	-89.072	negative
	yuc4685	Female	D	Mexico	21.051	-89.072	negative
	yuc4687	Male	D	Mexico	21.051	-89.072	negative
	yuc4689	Female	D	Mexico	21.051	-89.072	negative
MxYuTe15	yuc4693	Male	D	Mexico	21.051	-89.072	positive
	yuc4695	Female	D	Mexico	21.051	-89.072	negative
	yuc4697	Male	D	Mexico	21.051	-89.072	negative
	yuc4698	Female	D	Mexico	21.051	-89.072	negative
	yuc4699	Female	D	Mexico	21.051	-89.072	negative
	yuc4700	Female	D	Mexico	21.051	-89.072	negative
	yuc4701	Female	D	Mexico	21.051	-89.072	negative
	yuc4703	Female	D	Mexico	21.051	-89.072	negative
	yuc574	Female	D	Mexico	20.902	-87.4856	positive
	yuc83	Male		Mexico	20.902	-87.4856	negative
	yuc84	Male		Mexico	20.902	-87.4856	negative
	2B	Female	D	Belize	17.117	-89.1331	negative
	3B	Female	D	Belize	17.117	-89.1331	negative
	4B	Male	D	Belize	17.117	-89.1331	negative
	5B	Female	D	Belize	17.117	-89.1331	positive
	6B	Female	D	Belize	17.117	-89.1331	negative
	7B	Male	D	Belize	17.117	-89.1331	positive
BzCaCC05	13B	Female	D	Belize	17.117	-89.1331	positive
	14B	Female	D	Belize	17.117	-89.1331	negative
BzCaCC07	15B	Female	D	Belize	17.117	-89.1331	positive
	16B	Female	D	Belize	17.117	-89.1331	negative
	17B	Female	D	Belize	17.117	-89.1331	positive
BzCaCC09	18B	Male	D	Belize	17.117	-89.1331	positive
BzCaCC10	19B	Female	D	Belize	17.117	-89.1331	positive
	21B	Male	D	Belize	17.117	-89.1331	negative
	26B	Female	D	Belize	17.117	-89.1331	positive
	27B	Female	D	Belize	17.117	-89.1331	negative
	28B	Male	D	Belize	17.117	-89.1331	positive
	29B	Female	D	Belize	17.117	-89.1331	negative

Specimen ID: Specimens Used in Strain-typing	Specimen Lab ID	Sex	Ecotope, D = domestic, P = peridomestic, S = sylvan	Country	Latitude	Longitude	*T. cruzi* Infection
	46B	Female	D	Belize	16.266	-89.095	positive
	47B	Male	D	Belize	16.266	-89.095	positive
	48B	Male	D	Belize	16.266	-89.095	negative
	50B	Male		Belize	16.266	-89.095	negative
	51B	Female		Belize	16.266	-89.095	positive
	52B	Male		Belize	16.266	-89.095	positive
BzCaCC13	89B	Male		Belize	17.117	-89.1331	positive
BzCaCC14	90B	Female		Belize	17.117	-89.1331	positive
	91B	Male		Belize	17.117	-89.1331	negative
	93B	Male		Belize	17.117	-89.1331	positive
	94B	Male		Belize	17.117	-89.1331	negative
	95B	Male		Belize	17.117	-89.1331	negative
	96B	Male		Belize	17.117	-89.1331	negative
	97B	Male		Belize	17.117	-89.1331	negative
	99B	Female		Belize	16.266	-89.095	negative
	100B	Male		Belize	16.266	-89.095	positive
BzTDSJ04	107B	Female		Belize	16.266	-89.095	positive
	143B	Male		Belize	17.117	-89.1331	negative
	144B	Male		Belize	17.117	-89.1331	negative
	148B			Belize			negative
	216B	Female		Belize	16.266	-89.095	positive
Bz01	217B			Belize			positive
