# Supplementary material for: The diversity of the Chagas parasite, Trypanosoma cruzi, infecting the main Central American vector, Triatoma dimidiata, from Mexico to Colombia
Source: PLoS Negl Trop Dis. 2017 Sep 28;11(9):e0005878. doi: 10.1371/journal.pntd.0005878 (PMC5619707; doi:10.1371/journal.pntd.0005878)
Supplement: S2 Table — (PDF) [file pntd.0005878.s002.pdf]

Supplemental Table 2: Genbank 18S rDNA sequences from TcI isolates used in study

Country	Sample ID	Ecotope	Host	Genbank Accession No.
Brazil	TryCC 209	-	*Aotus sp.*	EU755220.1
Brazil	TryCC 331	-	*Cebus apella*	EU755222.1
Brazil	TryCC 125	-	*Didelphis aurita*	FJ549371.1
Brazil	TryCC 128	-	*Didelphis aurita*	FJ549372.1
Brazil	TryCC 1334	-	*Didelphis marsupialis*	EU755242.1
Brazil	TryCC 363	-	*Didelphis marsupialis*	FJ549375.1
Brazil	TryCC 711	-	*Didelphis marsupialis*	EU755229.1
Brazil	TryCC 1435	-	*Homo sapiens*	EU755245.1
Brazil	TryCC 1454	-	*Homo sapiens*	EU755250.1
Brazil	TryCC 743	-	*Homo sapiens*	EU755231.1
Brazil	TryCC 835	-	*Homo sapiens*	EU755233.1
Brazil	TryCC 971	-	*Homo sapiens*	EU755234.1
Brazil	TryCC 978	-	*Homo sapiens*	EU755235.1
Brazil	TryCC 1456	-	*Monodelphis brevicaudata*	FJ555623.1
Brazil	TryCC 884	-	*Panstrongylus megistus*	FJ549377.1
Brazil	TryCC 1094	-	*Philander frenata*	FJ549381.1
Brazil	TryCC 77	-	*Rhodnius brethesi*	EU755216
Brazil	TryCC 1073	-	*Rhodnius pictipes*	EU755236.1
Brazil	TryCC 1166	-	*Rhodnius pictipes*	EU755237.1
Brazil	TryCC 1359	-	*Rhodnius robustus*	EU755243.1
Brazil	TryCC 649	-	*Rhodnius robustus*	EU755225.1
Brazil	TryCC 1108	-	*Rhodnius stali*	FJ549382.1
Brazil	TryCC 1173	-	*Saguinus bicolor*	EU755238.1
Brazil	TryCC 1229	-	*Saguinus bicolor*	EU755240.1
Brazil	TryCC 1237	-	*Saguinus bicolor*	EU755241.1
Brazil	TryCC 269	-	*Saguinus midas*	EU755221.1
Brazil	TryCC 201	-	*Saimiri sciureus*	EU755219.1
Colombia	YDm1Bcl2	-	-	JF746668
Colombia	YAS1	Sylvan	*Alouatta* spp.	JF746669.1
Colombia	AADm1	Sylvan	*Didelphis marsupialis*	JF746675.1
Colombia	D1	Sylvan	*Didelphis marsupialis*	JF746683.1
Colombia	D16cl 8	Sylvan	*Didelphis marsupialis*	JF746667.1
Colombia	D18cl 8	Sylvan	*Didelphis marsupialis*	JF746666.1
Colombia	Dm11	Sylvan	*Didelphis marsupialis*	JF746680.1
Colombia	Dm38	Sylvan	*Didelphis marsupialis*	JF746681.1
Colombia	Dm7cl 6	Sylvan	*Didelphis marsupialis*	JF746682.1
Colombia	NDm1	Sylvan	*Didelphis marsupialis*	JF746673.1

Colombia	SLDm2cl	Sylvan	*Didelphis marsupialis*	JF746671.1
Colombia	SLDm2cl 9	Sylvan	*Didelphis marsupialis*	JF746671.1
Colombia	YDm1Mcl 2	Sylvan	*Didelphis marsupialis*	JF746678.1
Colombia	SLD1Ec cl 6	Sylvan	*Eratyrus cuspidatus*	JF746727.1
Colombia	CACQ	Domestic	*Homo sapiens*	JF746711.1
Colombia	CG	Domestic	*Homo sapiens*	JF746716.1
Colombia	DA cl 14	Domestic	*Homo sapiens*	JF746717.1
Colombia	DYR	Domestic	*Homo sapiens*	JF746704.1
Colombia	EEBB	Domestic	*Homo sapiens*	JF746714.1
Colombia	EH cl 1	Domestic	*Homo sapiens*	JF746715.1
Colombia	EM	Domestic	*Homo sapiens*	JF746713.1
Colombia	FcH cl2	Domestic	*Homo sapiens*	JF746721.1
Colombia	FEC	Domestic	*Homo sapiens*	JF746709.1
Colombia	JEM cl 2	Domestic	*Homo sapiens*	JF746707.1
Colombia	LCV	Domestic	*Homo sapiens*	JF746705.1
Colombia	LER cl 13	Domestic	*Homo sapiens*	JF746720.1
Colombia	LJVP cl 7	Domestic	*Homo sapiens*	JF746712.1
Colombia	MG	Domestic	*Homo sapiens*	JF746719.1
Colombia	SEV	Domestic	*Homo sapiens*	JF746708.1
Colombia	SMA	Domestic	*Homo sapiens*	JF746718.1
Colombia	SP	Domestic	*Homo sapiens*	JF746722.1
Colombia	XCh	Domestic	*Homo sapiens*	JF746706.1
Colombia	YLY cl 12	Domestic	*Homo sapiens*	JF746710.1
Colombia	NR1	Sylvan	*Rattus rattus*	JF746672.1
Colombia	Coy11 cl5	Sylvan	*Rhodnius colombiensis*	JF746724.1
Colombia	Necocli cl 6	Sylvan	*Rhodnius pallescens*	JF746698.1
Colombia	AAB3cl 2	Sylvan	*Rhodnius prolixus*	JF746694.1
Colombia	AAD6	Sylvan	*Rhodnius prolixus*	JF746685.1
Colombia	N5P14cl 7	Sylvan	*Rhodnius prolixus*	JF746702.1
Colombia	NA3	Sylvan	*Rhodnius prolixus*	JF746700.1
Colombia	NB2cl 5	Sylvan	*Rhodnius prolixus*	JF746699.1
Colombia	NC2	Sylvan	*Rhodnius prolixus*	JF746701.1
Colombia	SLA9	Sylvan	*Rhodnius prolixus*	JF746691.1
Colombia	SLB3	Sylvan	*Rhodnius prolixus*	JF746692.1
Colombia	SLD2	Sylvan	*Rhodnius prolixus*	JF746693.1
Colombia	SLF5	Sylvan	*Rhodnius prolixus*	JF746695.1
Colombia	SN5cl 7	Sylvan	*Rhodnius prolixus*	JF746723.1
Colombia	X1082cl 9	Domestic	*Rhodnius prolixus*	JF746689.1
Colombia	X1544cl 10	Domestic	*Rhodnius prolixus*	JF746688.1
Colombia	X236cl 8	Domestic	*Rhodnius prolixus*	JF746690.1
Colombia	YB1	Sylvan	*Rhodnius prolixus*	JF746687.1

Colombia	YD1	Sylvan	*Rhodnius prolixus*	JF746686.1
Colombia	Cepa 2cl 1 6	Sylvan	Rodent	JF746696.1
Colombia	Gal61cl 16	Sylvan	Rodent	JF746676.1
Colombia	SR2	Sylvan	Rodent	JF746674.1
Colombia	YTT1	Sylvan	*Tamandua tetradactyla*	JF746679.1
Colombia	TmPA1cl 6	Sylvan	*Triatoma maculata*	JF746728.1
Colombia	TV	Sylvan	*Triatoma venosa*	JF746729.1
Panama	R345	-	*Rhodnius pallescens*	FJ555617.1
Venezuela	Tc540	-	*Rattus rattus*	FJ555616.1

"-" = Information not available
