# Supplementary material for: The diversity of the Chagas parasite, Trypanosoma cruzi, infecting the main Central American vector, Triatoma dimidiata, from Mexico to Colombia
Source: PLoS Negl Trop Dis. 2017 Sep 28;11(9):e0005878. doi: 10.1371/journal.pntd.0005878 (PMC5619707; doi:10.1371/journal.pntd.0005878)
Supplement: S3 Table — (PDF) [file pntd.0005878.s003.pdf]

Supplemental Table 3: GenBank Spliced Leader – Intergenic Region (SL-IR) sequences from TcI isolates used in study

Country	Sample Name	Ecotope	Host	Genbank Accession No.
Argentina	Palda1cl9clone1	Sylvan	*Didelphis albiventris*	EF576830.1
Argentina	Tev91clone3	Domestic	*Triatoma infestans*	EF576831.1
Argentina	Tev91clone1	Domestic	*Triatoma infestans*	EF576832.1
Argentina	CHA	Domestic	*Homo sapiens*	FJ713366.1
Argentina	FRA	Domestic	*Homo sapiens*	FJ713371.1
Argentina	PA	Domestic	*Homo sapiens*	FJ713382.1
Argentina	PAVOOCl7	Domestic	*Triatoma infestans*	FJ713385.1
Argentina	Tev91Cl5	Domestic	*Triatoma infestans*	FJ713402.1
Argentina	VA	Domestic	*Homo sapiens*	FJ713404.1
Argentina	A-26b-motoHCl7	Domestic	*Canis familiaris*	GQ398789.2
Argentina	TALAVERDE	Domestic	*Triatoma infestans*	GQ398816.2
Argentina	PASCh	Domestic	*Homo sapiens*	GU179064.1
Argentina	K98	Domestic	*Homo sapiens*	GU179065.1
Argentina	CA-ICL72	Domestic	*Homo sapiens*	GU179066.1
Argentina	MIRANDACL78	Domestic	*Homo sapiens*	GU179067.1
Bolivia	P/209cl1clone1	Domestic	*Homo sapiens*	EF576816.1
Bolivia	P/209cl1clone4	Domestic	*Homo sapiens*	EF576817.1
Bolivia	P/209cl1clone7	Domestic	*Homo sapiens*	EF576818.1
Bolivia	P/11cl3 clone 1	Domestic	*Homo sapiens*	EF576839.1
Bolivia	P/217clone1	Domestic	*Homo sapiens*	EF576840.1
Bolivia	P/217clone2	Domestic	*Homo sapiens*	EF576841.1
Bolivia	S040clone1	Domestic	*Triatoma infestans*	EF576842.1
Bolivia	S040clone2	Domestic	*Triatoma infestans*	EF576843.1
Bolivia	86/2021 clone1	Sylvan	*Didelphis albiventris*	EF576844.1
Bolivia	AS	Domestic	*Homo sapiens*	FJ713356.1
Brazil	CUTIA	Sylvan	*Dasyprocta aguti*	AY367129.1
Brazil	G38.1clone1	Sylvan	*Didelphis albiventris*	EF576833.1
Brazil	G38.1clone2	Sylvan	*Didelphis albiventris*	EF576834.1
Brazil	*Trypanosoma cruzi*	-	*Human momia*	EF626693.1
Brazil	G	Sylvan	*Didelphis marsupialis*	FJ713372.1
Brazil	D11	Sylvan	*Didelphis marsupialis*	GU179068.1
Brazil	Sylvio	Domestic	*Homo sapiens*	X62674

Chile	TCC	Domestic	*Homo sapiens*	FJ713401.1
Chile	Gaj29cl8	Sylvan	*Meprai garjardoi*	GU903128.1
Chile	Gaj29cl6	Sylvan	*Meprai garjardoi*	GU903129.1
Chile	Sp130cl6	Sylvan	*Meprai spinolai*	GU903139.1
Chile	Sp130cl8	Sylvan	*Meprai spinolai*	GU903140.1
Chile	STPi1cl6	Sylvan	*Meprai spinolai*	GU903142.1
Chile	STPi1cl7	Sylvan	*Meprai spinolai*	GU903143.1
Chile	STPi1cl8	Sylvan	*Meprai spinolai*	GU903144.1
Chile	Til70cl6	Sylvan	*Meprai spinolai*	GU903145.1
Chile	Til70cl7	Sylvan	*Meprai spinolai*	GU903146.1
Chile	Til70cl8	Sylvan	*Meprai spinolai*	GU903147.1
Chile	WT cl6	Domestic	*Homo sapiens*	GU903155.1
Colombia	CGC	Domestic	*Homo sapiens*	AM259467.1
Colombia	JLC	Domestic	*Homo sapiens*	AM259468.1
Colombia	FChC	Domestic	*Homo sapiens*	AM259469.1
Colombia	DM28C	Sylvan	*Didelphis marsupialis*	AM259470.1
Colombia	SN6C	Domestic	*Rhodnius prolixus*	AM259471.1
Colombia	X380C	Domestic	*Rhodnius prolixus*	AM259472.1
Colombia	PALC	Sylvan	*Rhodnius prolixus*	AM259473.1
Colombia	EFC	Sylvan	*Triatoma dimidiata*	AM259474.1
Colombia	Td11C	Sylvan	*Triatoma dimidiata*	AM259475.1
Colombia	TVC	Peridomestic	*Triatoma venosa*	AM259476.1
Colombia	Mg10C	Sylvan	*Triatoma dimidiata*	AM259477.1
Colombia	JD1C	Sylvan	*Rhodnius prolixus*	AM259478.1
Colombia	TCIC	Sylvan	*Didelphis marsupialis*	AM259479.1
Colombia	RTDclone1	Sylvan	*Didelphis marsupialis*	EF576838.1
Colombia	JEMC	Domestic	*Homo sapiens*	EU127299.1
Colombia	SPC	Domestic	*Homo sapiens*	EU127300.1
Colombia	H135C	Peridomestic	*Canis familiaris*	EU127301.1
Colombia	H105C	Peridomestic	*Canis familiaris*	EU127302.1
Colombia	Dm38C	Sylvan	*Didelphis marsupialis*	EU127303.1
Colombia	Dm11C	Sylvan	*Didelphis marsupialis*	EU127304.1
Colombia	SN8C	Domestic	*Rhodnius prolixus*	EU127305.1
Colombia	X236	Domestic	*Rhodnius prolixus*	EU127306.1
Colombia	X150C	Domestic	*Rhodnius prolixus*	EU127307.1
Colombia	X1082C	Domestic	*Rhodnius prolixus*	EU127308.1
Colombia	X1084C	Domestic	*Rhodnius prolixus*	EU127309.1

Colombia	PRCC	Sylvan	*Rhodnius prolixus*	EU127310.1
Colombia	Mg11C	Peridomestic	*Rhodnius pallescens*	EU127311.1
Colombia	Td3C	Peridomestic	*Triatoma dimidiata*	EU127312.1
Colombia	HATC	Peridomestic	*Triatoma dimidiata*	EU127313.1
Colombia	EURC	Peridomestic	*Triatoma dimidiata*	EU127314.1
Colombia	G11C	Peridomestic	*Triatoma dimidiata*	EU127315.1
Colombia	EBE	Domestic	*Homo sapiens*	EU344771.1
Colombia	EMA	Domestic	*Homo sapiens*	EU344772.1
Colombia	MGC	Domestic	*Homo sapiens*	EU626722.1
Colombia	DAC	Domestic	*Homo sapiens*	EU626723.1
Colombia	NVC	Domestic	*Homo sapiens*	EU626724.1
Colombia	JVC	Domestic	*Homo sapiens*	EU626725.1
Colombia	EB2daC	Domestic	*Homo sapiens*	EU626726.1
Colombia	SEVC	Domestic	*Homo sapiens*	EU626727.1
Colombia	DYRC	Domestic	*Homo sapiens*	EU626728.1
Colombia	D12C	Sylvan	*Didelphis marsupialis*	EU626729.1
Colombia	H10C	Peridomestic	*Canis familiaris*	EU626730.1
Colombia	Rp523C	Sylvan	*Rhodnius prolixus*	EU626732.1
Colombia	N5P14C	Sylvan	*Rhodnius prolixus*	EU626733.1
Colombia	Rp513C	Sylvan	*Rhodnius prolixus*	EU626734.1
Colombia	Necocli C	Sylvan	*Rhodnius pallescens*	EU626735.1
Colombia	Coy11C	Sylvan	*Rhodnius colombiensis*	EU626736.1
Colombia	TdC	Peridomestic	*Triatoma dimidiata*	EU626737.1
Colombia	Mg9C	Peridomestic	*Triatoma dimidiata*	EU626738.1
Colombia	Cepa2	Sylvan	*Rhodnius colombiensis*	FJ463160.1
Colombia	Amp 7	Sylvan	*Panstrongylus geniculatus*	FJ713357.1
Colombia	Cas1	Domestic	*Rhodnius prolixus*	FJ713359.1
Colombia	Cas10	Sylvan	*Rhodnius prolixus*	FJ713360.1
Colombia	Cas15	Sylvan	*Rhodnius prolixus*	FJ713361.1
Colombia	Cas16	Peridomestic	*Rhodnius prolixus*	FJ713362.1
Colombia	Cas18	Sylvan	*Didelphis marsupialis*	FJ713363.1
Colombia	Cas19	Sylvan	*Rhodnius prolixus*	FJ713364.1
Colombia	Coy8	Sylvan	*Didelphis marsupialis*	FJ713367.1
Colombia	Coy9	Sylvan	*Rhodnius colombiensis*	FJ713368.1
Colombia	Fer1	Sylvan	*Rhodnius pallescens*	FJ713370.1
Colombia	Mg1	Sylvan	*Rhodnius pallescens*	FJ713377.1

Colombia	Mg11	Sylvan	*Rhodnius pallescens*	FJ713379.1
Colombia	MG	Domestic	*Homo sapiens*	FJ713380.1
Colombia	Put4	Sylvan	*Rhodnius robustus*	FJ713387.1
Colombia	Sebas16	Sylvan	*Rhodnius pallescens*	FJ713389.1
Colombia	SN11	-	*Rhodnius prolixus*	FJ713393.1
Colombia	SN12	Domestic	*Rhodnius prolixus*	FJ713394.1
Colombia	SO6	Sylvan	*Rhodnius pallescens*	FJ713395.1
Colombia	SP	Domestic	*Homo sapiens*	FJ713397.1
Colombia	STP3.3	Sylvan	*Rhodnius prolixus*	FJ713399.1
Colombia	W3534	Domestic	*Homo sapiens*	FJ713406.1
Colombia	Necocli2Cl1	Sylvan	*Rhodnius pallescens*	GU179078.1
Colombia	Coy12Cl5	Sylvan	*Rhodnius colombiensis*	GQ398803.2
Costa Rica	BOL	-	-	JQ028863.1
Costa Rica	JEN	-	-	JQ028864.1
French Guiana	VTH	Sylvan	*Homo sapiens*	FJ713405.1
Mexico	Tep23cl4clone1	Peridomestic	*Meccus longipennis*	EF576819.1
Mexico	Tep23cl4clone7	Peridomestic	*Meccus longipennis*	EF576820.1
Mexico	Tep23cl4clone16	Peridomestic	*Meccus longipennis*	EF576821.1
Mexico	JJOclone1	Domestic	*Homo sapiens*	EF576822.1
Mexico	JJOclone2	Domestic	*Homo sapiens*	EF576823.1
Mexico	Cari137clone1	Sylvan	*Meccus complex*	EF576824.1
Mexico	Cari137clone2	Sylvan	*Meccus complex*	EF576825.1
Mexico	Gue536clone1	Peridomestic	*Meccus longipennis*	EF576826.1
Mexico	Gue536clone2	Peridomestic	*Meccus longipennis*	EF576827.1
Mexico	L3033clone1	Sylvan	*Meccus longipennis*	EF576828.1
Mexico	L3033clone2	Sylvan	*Meccus longipennis*	EF576829.1
Mexico	Pla20clone3	Domestic	*Meccus picturata*	EF576835.1
Mexico	Pla20clone4	Domestic	*Meccus picturata*	EF576836.1
Mexico	H1clone1	Domestic	*Homo sapiens*	EF576845.1
Mexico	H1clone2	Domestic	*Homo sapiens*	EF576846.1
Mexico	Sba54clone1	Peridomestic	*Triatoma barberi*	EF576847.1
Mexico	Sba54clone2	Peridomestic	*Triatoma barberi*	EF576848.1
Mexico	800383 clone1	Domestic	*Meccus pallidipennis*	EF576849.1
Mexico	HD	Sylvan	-	FJ713374.1
Panama	Pan4cl4	Domestic	*Homo sapiens*	GU903133.1
Panama	Pan4cl5	Domestic	*Homo sapiens*	GU903134.1
Paraguay	V305Cl4	Domestic	*Triatoma infestans*	GQ398821.2
Paraguay	V75Cl7	Peridomestic	*Triatoma infestans*	GU179069.1
Paraguay	V75Cl1	Peridomestic	*Triatoma infestans*	GU903148.1
Paraguay	V75Cl2	Peridomestic	*Triatoma infestans*	GU903149.1
Paraguay	V75Cl5	Peridomestic	*Triatoma infestans*	GU903150.1

Paraguay	V75Cl6	Peridomestic	*Triatoma infestans*	GU903151.1
Paraguay	V305Cl3	Domestic	*Triatoma infestans*	GU903152.1
Paraguay	V305Cl6	Domestic	*Triatoma infestans*	GU903153.1
Paraguay	V305Cl7	Domestic	*Triatoma infestans*	GU903154.1
-	*Trypanosoma cruzi*	-	-	X00632.1
United States	Raccoon70clone2	Sylvan	*Procyon lotor*	EF576837.1
United States	USA1	Sylvan	*Triatoma gerstaeckeri*	GU179070.1
United States	USA108	Sylvan	*Triatoma gerstaeckeri*	GU179073.1
United States	USA111	Sylvan	*Triatoma gerstaeckeri*	GU179074.1
United States	USA115	Sylvan	*Triatoma gerstaeckeri*	GU179075.1
United States	BG08011	Sylvan	*Triatoma gerstaeckeri*	GU179076.1
United States	S29MHNOLA	-	-	KM376443.1
United States	S29MLNOLA	-	-	KM376444.1
United States	S6MLNOLA	-	-	KM376445.1
United States	S7MHNOLA	-	-	KM376446.1
United States	S9MSNOLA	-	-	KM376447.1
United States	S26MHNOLA	-	-	KM376448.1
United States	S6MMNOLA	-	-	KM376449.1
United States	Flop2	Sylvan	*Didelphis virginiana*	GU179077.1
Venezuela	2414	Sylvan	*Homo sapiens*	GU903123.1
Venezuela	EP	Domestic	*Homo sapiens*	GU903126.1
Colombia	-	-	-	EU626731.1

“-“ = Information not available
