# Supplementary figures and images for: The diversity of the Chagas parasite, Trypanosoma cruzi, infecting the main Central American vector, Triatoma dimidiata, from Mexico to Colombia
Source: PLoS Negl Trop Dis. 2017 Sep 28;11(9):e0005878. doi: 10.1371/journal.pntd.0005878 (PMC5619707; doi:10.1371/journal.pntd.0005878)

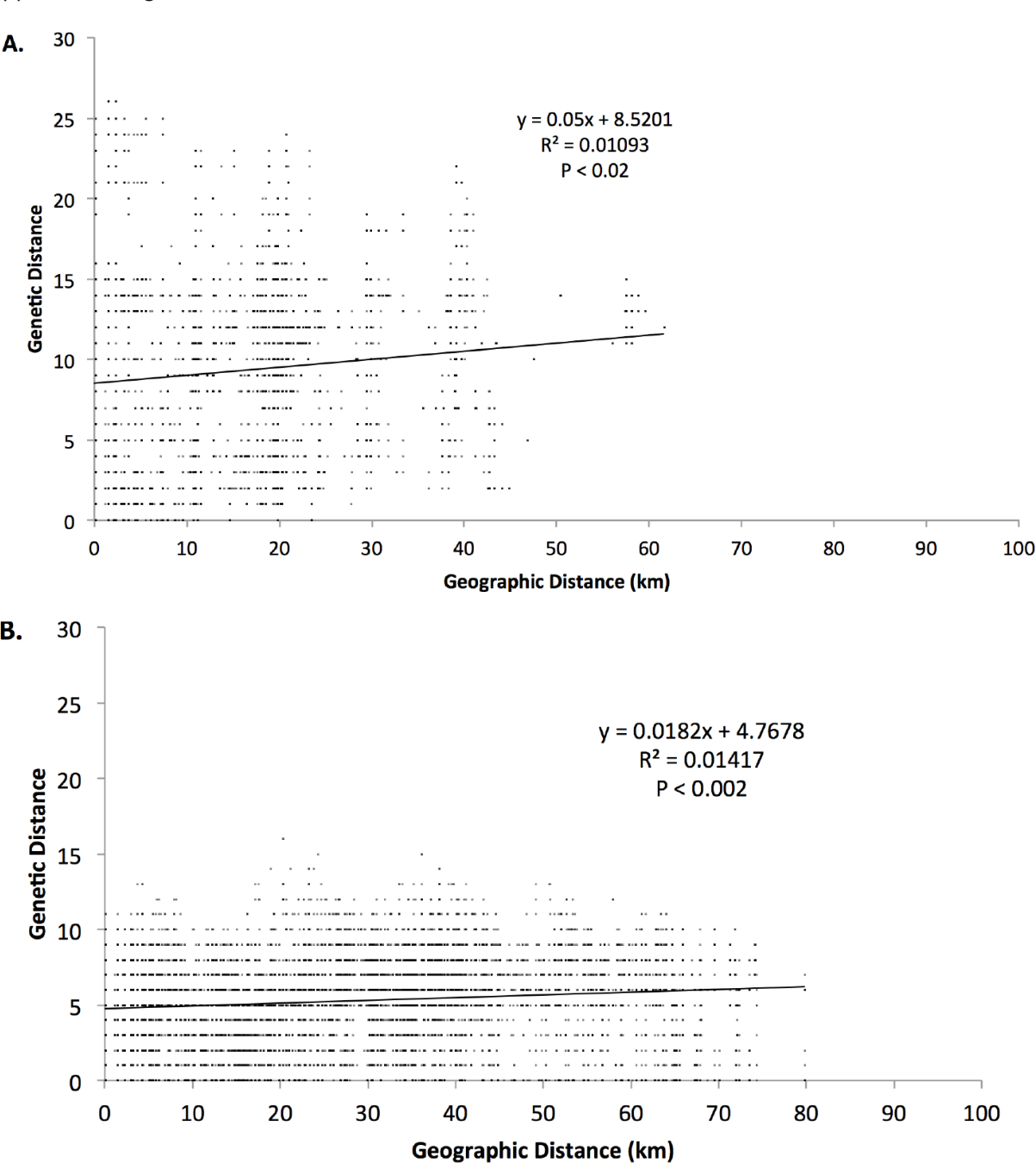

Supplement: S1 Fig — (TIF) [file pntd.0005878.s004.tif]
